# Molecular characterization of carbapenem-resistant Enterobacterales (CRE) and *in vitro* activity of novel beta lactams against CRE isolates from Malaysia

**DOI:** 10.1128/spectrum.00553-25

**Published:** 2025-09-22

**Authors:** Fairuz Abdul Rashid, Nurzam Suhaila Che Hussin, Nurul Fathiyah Zaipul Anuar, Noraziah Sahlan, Navindra Kumari Palanisamy, Fadzilah Mohd Nor

**Affiliations:** 1 Microbiology Unit, Department of Pathology, Hospital Kuala Lumpur 58983, Kuala Lumpur, Malaysia; 2 Department of Medical Microbiology & Parasitology, Faculty of Medicine, Universiti Teknologi MARA, Jalan Hospital 54703, Selangor, Malaysia; 3 Institute of Medical Molecular Biotechnology (IMMB), Faculty of Medicine, Universiti Teknologi MARA, Jalan Hospital 54703, Selangor, Malaysia; 4 Integrative Pharmacogenomics Research Institute (i-PROMISE), Level 7, Ff3, Faculty of Pharmacy, Universiti Teknologi MARA 54703, Selangor, Malaysia; Universidad de Buenos Aires, Buenos Aires, Argentina

**Keywords:** carbapenem-resistant Enterobacterales (CRE), carbapenemase genes, novel β-lactam agents, susceptibility, carbapenem

## Abstract

Knowledge gap on the susceptibility of novel β-lactam agents (cefiderocol, ceftazidime-avibactam, imipenem-cilastatin-relebectam, and aztreonam) against carbapenem-resistant Enterobacterales (CRE) has been recognized. This study aimed to genotypically characterize CRE isolates and investigate the novel β-lactam activity against CRE. CRE is defined as Enterobacterales that is phenotypically non-susceptible to any carbapenems, including imipenem, meropenem, and ertapenem. A total of 154 CRE isolates were collected from two tertiary centers in Malaysia from October 2023 to May 2024. Carbapenemase-producing genes (*bla*_NDM_, *bla*_OXA-48_, *bla*_KPC_, *bla*_VIM_, and *bla*_IMP_,) were detected using PCR. Susceptibility to β-lactams was determined using disc diffusion. Of 154 CRE isolates, 102 (66.2%) were carbapenemase-producing carbapenem-resistant Enterobacterales (CP-CRE). *bla*_NDM_ (76/102; 74.5%), *bla*_OXA-48-like_ (17/102; 16.7%), *bla*_NDM_ & *bla*_OXA-48-like_ (8/102; 7.8%), and *bla*_NDM_ & *bla*_VIM_ (1/102; 1.0%) were identified among the CP-CRE isolates. The proportion of CRE isolates that exhibited susceptibility towards cefiderocol, ceftazidime-avibactam, and imipenem-cilastatin-relebactam was 86.4% (133/154), 41.6% (64/154), and 26.0% (40/154), respectively. Among *bla*_NDM_-harboring isolates, cefiderocol (57/76; 75.0%) demonstrated superior activity compared with ceftazidime-avibactam (3/76; 3.9%) and imipenem-cilastatin-relebectam (1/76; 1.3%). Among isolates harboring *bla*_OXA-48-like_, cefiderocol, ceftazidime-avibactam, and imipenem-cilastatin-relebectam demonstrated 100% (17/17), 70.6% (12/17), and 17.6% (3/17) susceptibility, respectively. Nine isolates that harbored two genes (eight *bla*_NDM_ + *bla*_OXA-48-like_, one *bla*_NDM_ + *bla*_VIM_) demonstrated 100% susceptibility to cefiderocol but 100% resistance to ceftazidime-avibactam and imipenem-cilastatin-relebectam. The ceftazidime-avibactam plus aztreonam combination achieved 100% susceptibility in isolates harboring metallo-β-lactamases-producing genes; *bla*_NDM_ (76/76; 100%), *bla*_NDM_ + *bla*_OXA-48-like_ (8/8; 100%), and *bla*_NDM_ + *bla*_VIM_ (1/1; 100%). *bla*_NDM_ was the most prevalent gene causing CRE. Cefiderocol has the greatest activity compared with other investigated β-lactams.

**IMPORTANCE:**

Carbapenem-resistant Enterobacterales (CRE) has been recognized as a priority and public health concern requiring urgent attention for the development of effective antimicrobial resistance (AMR) prevention and control strategies. Differentiating between carbapenemase-producing CRE (CP-CRE) and non-CP-CRE, along with identifying carbapenemase-producing genes, is essential for guiding targeted antibiotic therapy. Among novel β-lactam agents, cefiderocol and the combination of ceftazidime-avibactam and aztreonam have shown promising activity against *bla*_NDM_-producing CRE, supporting precision medicine approaches. Nevertheless, our study observed the emergence of cefiderocol resistance in isolates without prior drug exposure, highlighting a potential challenge in combating AMR.

## INTRODUCTION

Antimicrobial resistance (AMR) is no longer a silent pandemic with a forecast of 10 million deaths worldwide in 2050 (1). To date, carbapenem-resistant Enterobacterales (CRE) is placed first in the 2024 list of antibiotic-resistant bacterial priority pathogens by the World Health Organization (WHO). CRE comprises a heterogeneous group of Enterobacterales that is responsible for various infection syndromes, including but not limited to bloodstream, respiratory tract, intra-abdominal, and urinary tract infections (2).

In CRE, the antibiotic resistance may be caused by multiple potential mechanisms, broadly divided into those that are carbapenemase-producing and non-carbapenemase-producing. Production of carbapenemases is the primary mechanism for the antibiotic resistance among CRE strains. Carbapenemases are β-lactamases using carbapenems as hydrolysis substrates, and at present, acquired Ambler class A *Klebsiella pneumoniae* carbapenemases (KPC), class B metallo-β-lactamases (MBL) New Delhi metallo-β-lactamases (NDM), Verona integron-encoded metallo-β-lactamases (VIM), imipenem-hydrolyzing metallo-β-lactamases (IMP), and class D oxacillinases (eg. OXA-48, OXA-181) are identified. These are the most important determinants sustaining resistance to carbapenems (3). Carbapenemase-producing CRE (CP-CRE) is of major clinical and public health concern, owing to the highest estimated burden among all multi-drug-resistant (MDR) Gram-negative bacteria and their widespread prevalence with resistance at an alarming pace (4).

The CRE infections are complex to manage due to limited treatment options, resulting in a substantial health and economic burden, which further highlights the need for novel and innovative treatment options along with good preventive and control measures (5). Furthermore, since 2017, more than 80% of approved antibiotics for CRE infections have demonstrated a limited degree of novel activity, with these agents being derivatives of already known antibiotic classes to which multiple resistance mechanisms exist (6). Thus, this adds up to the challenges and complexity of the AMR.

In recent years, new antibiotics and novel combination agents, including β-lactam inhibitors with carbapenems or cephalosporins, have entered the pipeline, mainly targeting Class A and some Class D enzymes. However, only a few novel agents are available in the market that can also inhibit Class B enzymes, such as cefiderocol, a siderophore cephalosporin, which displays *in vitro* activity against all isolates harboring β-lactamases (6). The updated clinical guidelines have recommended targeted management of CRE infection based on the recently approved agents in the antibiotic pipeline (7, 8). The Infectious Diseases Society of America (IDSA) strongly advocates carbapenemase phenotypic and/or genotypic testing to be pursued by all clinical microbiology laboratories to inform optimal treatment for CRE infections. Recently, ceftazidime-avibactam in combination with aztreonam, or cefiderocol as monotherapy, has been recommended by the IDSA as the treatment option for infections caused by CP-CRE (NDM and other Class B enzymes). Besides that, ceftazidime-avibactam is the preferred treatment option for infections caused by Class A and Class D CP-CRE.

Nonetheless, polymyxin B and colistin (polymyxin E) are widely used to treat infections caused by CRE in Malaysia. These polypeptide antibiotics, previously considered as the last resort for infections caused by MDR Gram-negative bacteria, are no longer the treatment of choice for CRE infections (7, 8).

The susceptibility profile of recently approved β-lactam antibiotics against CRE has not been well-described in Malaysia. With a global shift in CRE treatment from polypeptide antibiotics to novel β-lactam agents, understanding their efficacy is crucial. Determining the antibiotic susceptibility pattern, alongside identifying carbapenemase-producing genes, is essential for optimizing treatment strategies. In this study, we genotypically characterized a cohort of consecutive CRE isolates from two Malaysian centers and evaluated *in vitro* activity of novel β-lactam agents (cefiderocol, ceftazidime-avibactam plus aztreonam, and imipenem-cilastatin-relebactam) against these isolates.

## MATERIALS AND METHODS

### Bacterial isolates

A total of 154 clinical CRE isolates were collected from diagnostic microbiology laboratories of two hospitals: Hospital Kuala Lumpur (*n* = 137) and Hospital Al-Sultan Abdullah (HASA), Universiti Teknologi MARA (UiTM), Puncak Alam (*n* = 17), between October 2023 and May 2024. The isolates were obtained from sterile and non-sterile specimens, including blood, cerebrospinal fluid (CSF), pleural fluid, peritoneal fluid, bone, bone marrow, pus aspirate/swab, sputum, tracheal aspirate, bronchial alveolar lavage, and urine. Bacterial identification was performed using matrix-assisted laser desorption/ionization time-of-flight mass spectrometry (MALDI-TOF MS) (Bruker Daltonics, Billerica, USA) and the VITEK 2 system (bioMérieux, Inc., Durham, USA). The CRE isolates from pure cultures or mixed cultures of two organisms were included, while repeated isolates of the same species from the same patient were excluded to minimize bias and ensure representative data. All isolates were stored at −80°C in glycerol for further testing.

### Antimicrobial susceptibility testing (AST)

The CRE is defined as Enterobacterales isolate that is phenotypically non-susceptible to any one of the carbapenems, including ertapenem, meropenem, and imipenem, according to breakpoints by the Clinical Laboratory and Standards Institute (CLSI) M100 Performance Standards for Antimicrobial Susceptibility Testing, 34th edition (9). A gradient diffusion method, E-test (bioMérieux, Inc. Durham, USA), was performed on the CRE isolates to determine the susceptibility towards carbapenems.

Antibiotic susceptibility testing for ceftazidime-avibactam (30/20 µg), imipenem-cilastatin-relebactam (10/25 µg), cefiderocol (30 µg), and aztreonam (30 µg) was performed using the Kirby-Bauer disk diffusion method (Liofilchem, Teramo, Italy). A bacterial suspension adjusted to a 0.5 McFarland standard was lawned on Mueller-Hinton agar, followed by placement of antibiotic disks and incubation at 35°C ± 2°C for 18 to 24 h. E-test was used to confirm ceftazidime-avibactam susceptibility. Results were interpreted according to CLSI 2024 breakpoints (Table 1) (9).

**TABLE 1:** Zone diameter and MIC breakpoints for Enterobacterales according to Clinical and Laboratory Standards Institute (CLSI) 2024 Guidelines

Antibiotic	Disk content	Interpretive categories and zone diameter breakpoints, nearest whole mm			Interpretive categories and MIC breakpoints, μg/mL^*c*^		
		S	I	R	S	I	R
Cefiderocol	30 µg	≥ 16	9–15	≤ 8			
Ceftazidime-avibactam^*a*^	30/20 µg	≥ 21	–^*b*^	≤ 20	≤ 8/4	–	≥ 16/4
Imipenem-cilastatin-relebactam	10/25 µg	≥ 25	21–24	≤ 20			
Aztreonam	30 µg	≥ 21	18–20	≤ 17			

^*a*^ For ceftazidime-avibactam, confirmatory MIC testing is indicated for isolates with zones of 20–22 mm to avoid reporting false-susceptible or false-resistant results.

^*b*^ “–”, not available.

^*c*^ The grey shade indicate that the MIC test was not performed as the antibiotic breakpoints using disk diffusion are susceptible.

### *In vitro* synergistic activity of ceftazidime-avibactam combined with aztreonam against metallo-β-lactamases-producing isolates

The synergistic activity of the combination between ceftazidime-avibactam and aztreonam was assessed using the ceftazidime-avibactam plus aztreonam broth disk elution method as per recommendation by CLSI M100 guideline (9). A bacterial suspension adjusted to a 0.5 McFarland standard is inoculated into four tubes of cation-adjusted Mueller-Hinton broth (CAMHB). Antibiotic disks (Liofilchem, Teramo, Italy) were added into three of the CAMHB tubes, one received a 30 µg aztreonam disk, another a 30/20 µg ceftazidime-avibactam disk, and the third tube had a combination of both ceftazidime-avibactam and aztreonam disks. While the fourth tube, which served as the growth control, contained no antibiotic disk. The inoculated tubes were incubated at 33°C to 35°C in ambient air for 16 to 20 h. Following incubation, the tubes were examined for turbidity. A clear broth interpreted as susceptibility, while presence of turbidity indicated resistance.

### Detection of genes encoding carbapenemase production (*bla*_NDM_, *bla*_OXA-48_, *bla*_KPC_**,** *bla*_VIM_, and *bla*_IMP_) using PCR

All isolates were subjected to detection of carbapenemase-producing genes (*bla*_NDM_, *bla*_OXA-48_, *bla*_KPC_, *bla*_VIM_, and *bla*_IMP_) using multiplex conventional polymerase chain reaction (PCR). Genomic DNA was extracted using the boiling method. A few colonies grown on blood agar were suspended in 200 µL of distilled water, heated at 95°C for 10 min, and centrifuged at 10,000 rpm for another 10 min. One microliter of the supernatant was used as a DNA template for PCR.

The primers used in this study were adapted from a previous study (10). The positive control isolates for multiplex PCR included ATCC BAA-1705 *Klebsiella pneumoniae* (*bla*_KPC_) and ATCC BAA-2146 *Klebsiella pneumoniae* (*bla*_NDM_). Additionally, reference strains obtained from the National Institute of Health, Malaysia, included *Klebsiella pneumoniae* (2503/2021-*bla*_OXA-48-like_), *Enterobacter hormaechei* (2238/23-*bla*_VIM_), and *Klebsiella pneumoniae* (2477/23-*bla*_IMP_).

A primer targeting 16S rRNA served as an internal control for each PCR reaction. The PCR amplification conditions were as follows: an initial denaturation at 95°C for 2 min; followed by 35 cycles of denaturation at 95°C for 30 s, annealing at 63°C for 30 s, and extension at 72°C for 1 min; then a final extension at 72°C for 3 min in a Veriti thermocycler (Thermo Fisher Scientific, Waltham, USA). PCR products were analyzed on a 2% agarose gel stained with SYBR Safe DNA gel stain (Thermo Fisher Scientific, Waltham, USA) and visualized under UV light. Isolates were classified as CP-CRE if at least one carbapenemase gene was detected by PCR.

The Xpert Carba-R assay (Cepheid, Sunnyvale, USA), a qualitative *in vitro* multiplex real-time PCR assay, was used to validate the detection of carbapenemase genes (*bla*_NDM_, *bla*_OXA-48_, *bla*_KPC_, *bla*_VIM_, and *bla*_IMP_). The validation was performed using the five positive control isolates and 30 randomly selected CP-CRE and non-CP-CRE isolates.

### Statistical methods

Descriptive statistics for patient age, gender, and department were calculated using the median for continuous variables and frequency counts (percentages) for categorical variables, as appropriate. Comparisons between groups based on carbapenemase-producing genes were performed using the Kruskal-Wallis H test for continuous variables. For categorical variables, the Pearson χ² test was used, while Fisher’s exact test was applied when expected cell counts were ≤5. All tests were two-tailed, with statistical significance set at *P* ≤ 0.05. Data analysis was conducted using IBM SPSS Statistics version 29.0.

## RESULTS

### Clinical characteristics of CRE isolates

Among the identified bacterial species, *Klebsiella pneumoniae* was the most prevalent (87/154; 56.5%), followed by the *Enterobacter cloacae* complex (36/154; 23.4%) and *Escherichia coli* (21/154; 13.6%). Other CRE identified were *Citrobacter freundii*, *Citrobacter koseri*, *Klebsiella aerogenes*, *Klebsiella oxytoca*, *Morganella morganii*, and *Proteus mirabilis*. The most common sources of bacterial isolation were urine (57/154; 37.0%), blood (38/154; 24.7%), tracheal aspirate (17/154; 11.0%), sputum (11/154; 7.1%), and pus swabs or aspirates (11/154; 7.1%). CRE isolates were primarily recovered from general wards (103/154; 66.9%), followed by high-dependency and intensive care units (36/154; 23.4%), with a smaller proportion gathered from outpatient settings (15/154; 9.7%) (Table 2).

**TABLE 2:** Demographic features of patients and CRE isolates

Features	No. of isolates (%)	CP-CRE	Non-CP-CRE
	(*n* = 154)	(*n* = 102; 66.2%)	(*n* = 52; 33.8%)
Organism			
*Klebsiella pneumoniae*	87 (56.5%)	60 (69.0%)	27 (31.0%)
*Enterobacter cloacae complex*	36 (23.4%)	18 (50.0%)	18 (50.0%)
*Escherichia coli*	21 (13.6%)	16 (76.2%)	5 (23.8%)
Others	10 (6.5%)	8 (80.0%)	2 (20.0%)
Specimen			
Urine	57 (37.0%)	45 (78.9%)	12 (21.1%)
Blood	38 (24.7%)	26 (68.4%)	12 (31.6%)
Tracheal aspirate	17 (11.0%)	11 (64.7%)	6 (35.3%)
Sputum	11 (7.1%)	6 (54.5%)	5 (45.5%)
Pus swab/aspirate	11 (7.1%)	4 (36.4%)	7 (63.6%)
Others	20 (13.0%)	10 (50.0%)	10 (50.0%)
Department			
General ward	103 (66.9%)	69 (67.0%)	34 (33.0%)
High dependency/Intensive care	36 (23.4%)	22 (61.1%)	14 (38.9%)
Outpatient	15 (9.7%)	11 (73.3%)	4 (26.7%)

### Detection of genes encoding carbapenemase production

Of the 154 CRE isolates, 102 (66.2%) were carbapenemase-producing CRE (CP-CRE), while 52 (33.8%) were non–CP-CRE (Table 2). Among CP-CRE isolates, *Klebsiella pneumoniae* was the most frequently identified species (60/102; 58.8%), followed by the *Enterobacter cloacae* complex (18/102; 17.6%) and *Escherichia coli* (16/102; 15.7%). A similar species distribution was observed among non-CP-CRE isolates: *Klebsiella pneumoniae* (27/52; 51.9%), *Enterobacter cloacae* complex (18/52; 34.6%), and *Escherichia coli* (5/52; 9.6%). Of the 102 CP-CRE isolates, *bla*_NDM_ was the most prevalent carbapenemase gene (76/102; 74.5%) (Table 3), followed by *bla*_OXA-48-like_ (17/102; 16.7%). While remaining nine CP-CRE isolates (9/102; 8.8%) co-harbored more than one carbapenemase gene: *bla*_NDM_ and *bla*_OXA-48-like_ (8/102; 7.8%) and *bla*_NDM_ and *bla*_VIM_ (1/102; 1.0%). None of the isolates in this study were found to harbor the *bla*_KPC_ or *bla*_IMP_ carbapenemase genes.

**TABLE 3:** Distribution of carbapenemase-producing genes according to CRE species

Organism	No. of CP-CRE, *n* = 102	*bla*_**NDM**_	*bla*_**OXA-48-like**_	*bla*_NDM_ &*bla*_**OXA-48-like**_	*bla*_NDM_ &*bla*_**VIM**_
	(*n* = 102)	(*n* = 76; 74.5%)	(*n* = 17, 16.7%)	(*n* = 8; 7.8%)	(*n* = 1;1.0%)
*Klebsiella pneumoniae*	60 (58.8%)	44 (73.3%)	11 (18.3%)	5 (8.3%)	0 (0.0%)
*Enterobacter cloacae complex*	18 (17.6%)	12 (66.7%)	3 (1.7%)	2 (1.1%)	0 (0.0%)
*Escherichia coli*	16 (15.7%)	15 (93.7%)	1 (6.3%)	0 (0.0%)	0 (0.0%)
Others	8 (7.8%)	5 (62.5%)	2 (2.5%)	1 (1.3%)	1 (1.3%)

### CRE susceptibility pattern to carbapenem

**Fig 1:**
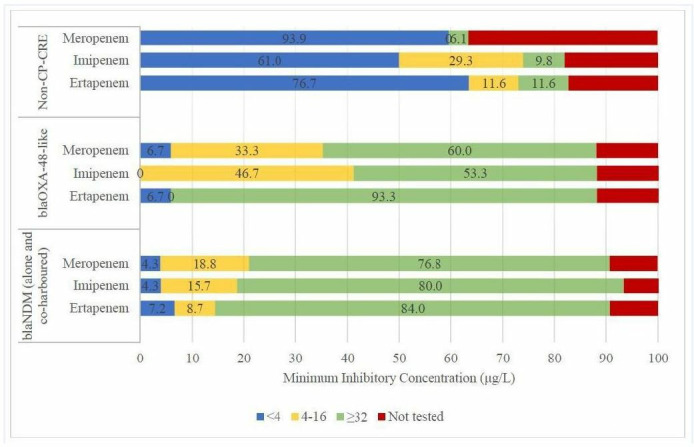
Distribution of carbapenem MICs among CP-CRE and non-CP-CRE isolates. Numbers represent percentages of all tested isolates for that antimicrobial and do not take into account non-tested isolates. Results of the Kruskal–Wallis H test indicated that there is a statistically significant difference in ertapenem, imipenem, and meropenem MICs between CP-CRE and non-CP-CRE isolates, respectively; *H*(4) = 75.249, *P* ≤ 0.001; *H*(4) = 67.068, *P* ≤ 0.001; *H*(4) = 69.146, *P* ≤ 0.001. *P*-value < 0.05 was considered statistically significant.

The association between different CRE genotypes and the minimum inhibitory concentrations (MICs) of carbapenems (ertapenem, meropenem, and imipenem) revealed statistically significant differences (*P* < 0.001) between CP-CRE and non-CP-CRE isolates. The MIC profiles of isolates harboring *bla*_NDM_ alone and those co-harboring *bla*_VIM_ and *bla*_OXA-48-like_ were analyzed together, as they exhibited similar resistance patterns (Fig. 1).

In CP-CRE isolates with *bla*_NDM_ alone or in combination with *bla*_VIM_ and *bla*_OXA-48-like_, the most frequently observed MICs were ≥32 µg/L for ertapenem (58/69; 84.0%), ≥32 µg/L for imipenem (56/70; 80.0%), and ≥32 µg/L for meropenem (53/69; 76.8%). Similarly, isolates harboring *bla*_OXA-48-like_ showed high MIC values, with ertapenem (≥32 µg/L in 14/15; 93.3%), imipenem (≥32 µg/L in 8/15; 53.3%), and meropenem (≥32 µg/L in 9/15; 60.0%). These findings indicate a consistent pattern of high MICs across all carbapenems for carbapenemase-producing genes.

Conversely, non-CP-CRE isolates exhibited lower MIC values, with the most frequently observed MICs being <4 µg/L for ertapenem (33/43; 76.7%), <4 µg/L for imipenem (25/41; 61.0%), and <4 µg/L for meropenem (31/33; 93.9%). The MIC analysis of imipenem has excluded *Morganella* spp. and *Proteus* spp., as these species may exhibit elevated MICs due to mechanisms other than carbapenemase production.

### Antimicrobial susceptibility testing and interpretation

**Fig 2:**
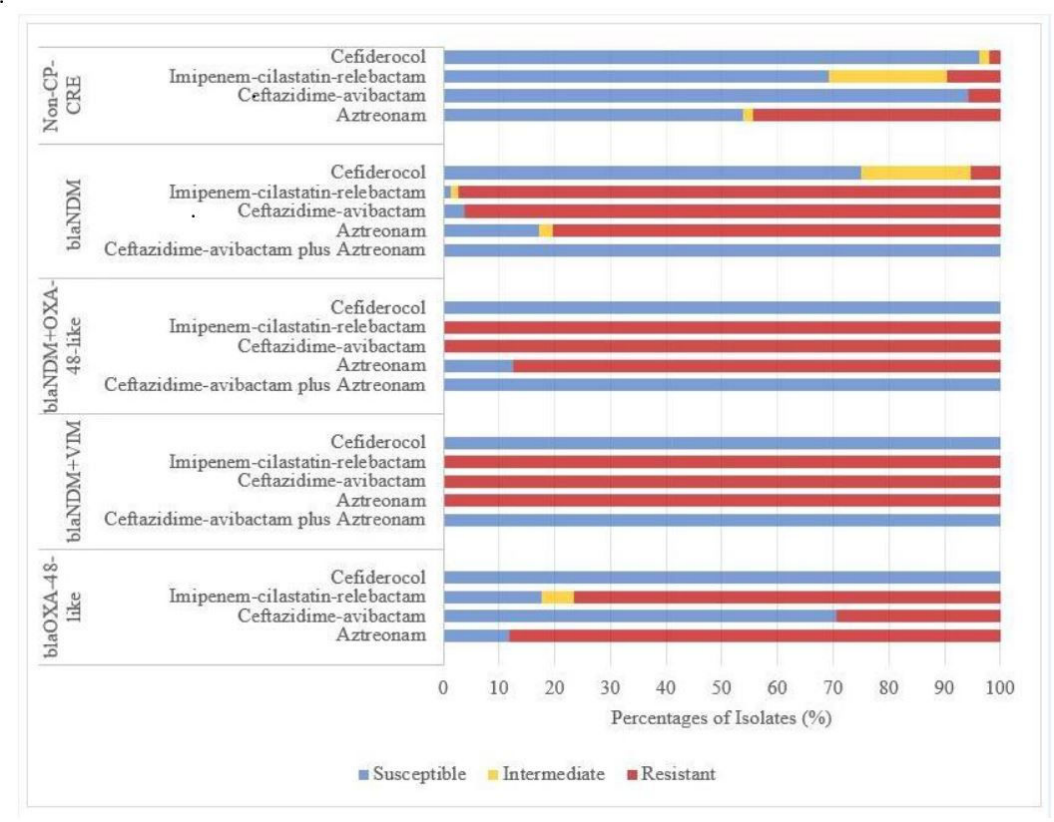
Distribution of β-lactam antibiotics susceptibility among CP-CRE and non-CP-CRE isolates.

Susceptibility testing of 154 CRE isolates to β-lactam agents showed the following rates: cefiderocol (86.4%), ceftazidime-avibactam (41.6%), imipenem-cilastatin-relebactam (26.0%), and aztreonam (28.6%). Overall, all tested antibiotics demonstrated greater activity against non-CP-CRE than CP-CRE isolates (Fig. 2).

Among the 102 CP-CRE isolates, cefiderocol exhibited the highest activity (75.0%) against 76 isolates harboring *bla*_NDM_ in contrast to ceftazidime-avibactam and imipenem-cilastatin-relebactam, which showed susceptibility rates of 3.9% and 1.3%, respectively (Table 4). Of 17 isolates harboring *bla*_OXA-48-like_, cefiderocol, ceftazidime-avibactam, and imipenem-cilastatin-relebactam exhibited susceptibility rates of 100%, 70.6%, and 17.6%, respectively. Meanwhile, all nine isolates co-harboring multiple carbapenemase genes (*bla*_NDM_ + *bla*_OXA-48-like_ in eight isolates, *bla*_NDM_ + *bla*_VIM_ in one isolate) were fully susceptible to cefiderocol (100%) but completely resistant to ceftazidime-avibactam and imipenem-cilastatin-relebactam (100%). Pairwise comparisons showed statistically significant differences (*P* < 0.05) in antibiotic susceptibility among *bla*_NDM_ - harboring isolates. Significant differences were also observed in ceftazidime-avibactam susceptibility among isolates harboring *bla*_OXA-48-like_ and those co-harboring *bla*_NDM_ + *bla*_OXA-48-like_ compared with other isolates. Imipenem-cilastatin-relebactam susceptibility testing did not include *Morganella* spp. and *Proteus* spp., as these species may exhibit elevated MICs due to non-carbapenemase mechanisms.

**TABLE 4:** Susceptibility profiles of β-lactam antibiotics against CRE isolates

Isolate group/Genotype	Antibiotic	Susceptible, n (%)	Intermediate, n (%)	Resistant, n (%)	*P*-value
CRE (*n* = 154)	Cefiderocol	133 (86.4%)	16 (10.3%)	5 (3.2%)	
	Ceftazidime-avibactam	64 (41.6%)	–^*c*^	90 (58.4%)	
	Imipenem-cilastatin-relebactam	40 (26.0%)	13 (8.4%)	101 (65.6%)	
	Aztreonam	44 (28.6%)	3 (1.9%)	107 (69.5%)	
CP-CRE (*n* = 102)	Cefiderocol	83 (81.4%)	15 (14.7%)	4 (3.9%)	0.011^*a*^
	Ceftazidime-avibactam	15 (14.7%)	–	87 (85.3%)	<0.001^*a*^
	Imipenem-cilastatin-relebactam	4 (3.9%)	2 (2.0%)	96 (94.1%)	<0.001^*a*^
	Aztreonam	16 (15.7%)	2 (2.0%)	84 (82.4%)	<0.001^*a*^
*bla*_NDM_ (*n* = 76)	Cefiderocol	57 (75.0%)	15 (19.7%)	4 (5.3%)	<0.001^*a*^
	Ceftazidime-avibactam	3 (3.9%)	–	73 (96.1%)	<0.001^*a*^
	Imipenem-cilastatin-relebactam	1 (1.3%)	1 (1.3%)	74 (97.4%)	<0.001^*a*^
	Aztreonam	13 (17.1%)	2 (2.6%)	61 (80.3%)	0.002^*a*^
*bla*_OXA-48-like_ (*n* = 17)	Cefiderocol	17 (100%)	0 (0%)	0 (0%)	0.131^*b*^
	Ceftazidime-avibactam	12 (70.6%)	–	5 (29.4%)	0.010^*a*^
	Imipenem-cilastatin-relebactam	3 (17.6%)	1 (5.9%)	13 (76.5%)	0.562^*b*^
	Aztreonam	2 (11.8%)	0 (0%)	15 (88.2%)	0.154^*b*^
*bla*_NDM_ *& bla*_OXA-48-like_ (*n* = 8)	Cefiderocol	8 (100%)	0 (0%)	0 (0%)	0.599^*b*^
	Ceftazidime-avibactam	0 (0%)	–	8 (100%)	0.021^*b*^
	Imipenem-cilastatin-relebactam	0 (0%)	0 (0%)	8 (100%)	0.113^*b*^
	Aztreonam	1 (12.5%)	0 (0%)	7 (87.5%)	0.441^*b*^
*bla*_NDM_ *& bla*_VIM_ (*n* = 1)	Cefiderocol	1 (100%)	0 (0%)	0 (0%)	1.000^*b*^
	Ceftazidime-avibactam	0 (0%)	–	1 (100%)	1.000^*b*^
	Imipenem-cilastatin-relebactam	0 (0%)	0 (0%)	1 (100%)	1.000^*b*^
	Aztreonam	0 (0%)	0 (0%)	1 (100%)	1.000^*b*^
Non-CP-CRE (*n* = 52)	Cefiderocol	50 (96.2%)	1 (1.9%)	1 (1.9%)	0.011^*a*^
	Ceftazidime-avibactam	49 (94.2%)	–	3 (5.8%)	<0.001^*a*^
	Imipenem-cilastatin-relebactam	36 (69.2%)	11 (21.2%)	5 (9.6%)	<0.001^*a*^
	Aztreonam	28 (53.8%)	1 (1.9%)	23 (44.3%)	<0.001^*a*^

^*a*^ Pearson Chi-Square test.

^*b*^ Fisher’s Exact test.

^*c*^ “–”, not available.

Aztreonam alone had limited activity against MBL-producing CRE isolates. However, *in vitro* synergy between ceftazidime-avibactam and aztreonam assessed using the disk elution method showed 100% (85/85) susceptibility among *bla*_NDM_-harboring, *bla*_NDM_ + *bla*_OXA-48-like_ and *bla*_NDM_ + *bla*_VIM_ - harboring isolates.

## DISCUSSION

Phenotypic antibiotic susceptibility testing and molecular identification of carbapenemase-producing genes are key components of a precision medicine approach in managing CRE infections. Differentiating between CP-CRE and non-CP-CRE is clinically important for guiding antibiotic selection. Based on this study, CP-CRE accounted for 66.2% of the 154 CRE isolates, slightly lower than the national prevalence of 77.5% reported by the National Institute of Health, Malaysia (11). CP-CRE resistance is mediated by carbapenemase production, which hydrolyzes carbapenems. These enzymes are encoded by carbapenemase-producing genes located on mobile genetic elements, such as plasmids or transposons, facilitating both vertical transmission within clonal lineages and horizontal transfer between different strains and species (12, 13).

Identifying the specific carbapenemase is crucial, as certain newer β-lactam antibiotics are active against specific carbapenemases. *bla*_NDM_ was the most frequently identified carbapenemase in the present study, consistent with findings from other centers in Malaysia and several Asian countries, including Thailand, India, and Egypt (11, 14–16).

In contrast, *bla*_KPC_ is more prevalent than *bla*_NDM_ in the United States, Southern Europe, and China (17–19). The variation in carbapenemase prevalence across countries and regions has been proposed to be attributed to factors including the nature of the primary reservoir favoring specific resistance genes, genetic structures of carbapenemase genes that enhance plasticity and mobility, selective pressure for resistance, and the level of human population exchange once a reservoir is established (20, 21).

Previously, CRE was predominantly associated with hospital-acquired infections. However, in recent years, reports have indicated the dissemination of CRE within the community (22, 23). In our study, 60.0% (9/15) of CRE isolates from outpatient settings harbored *bla*_NDM_, while 13.3% (2/15) carried *bla*_OXA-48-like_, and 6.7% (1/15) co-harbored *bla*_NDM_ + *bla*_OXA-48-like_, suggesting potential spread beyond healthcare settings.

The association between carbapenemase genes and carbapenem susceptibility in this study corresponds to previous reports (24). For instance, meropenem MICs ≥ 32 µg/mL were observed in 60%–100% of CP-CRE isolates, compared with only 6.1% in CRE isolates without detectable carbapenemase genes. This finding highlights the aggressiveness and increased ability of CP-CRE isolates in hydrolyzing carbapenems in contrast to non-CP-CRE.

Among all the novel β-lactams investigated in this study, cefiderocol demonstrated the greatest *in vitro* activity against CRE isolates, regardless of carbapenemase production. Similar findings were observed when specific carbapenemase-producing genes were analyzed. In this study, cefiderocol exhibited 75% susceptibility against *bla*_NDM_-harboring isolates, in comparison to other tested novel β-lactams. Moreover, all nine CRE isolates co-harboring *bla*_NDM_ with another carbapenemase-producing gene were fully (100%) susceptible to cefiderocol.

Cefiderocol, a novel siderophore cephalosporin, is a synthetic conjugate that binds to iron and enters bacterial cells via active iron transporters. Its antibacterial effect is primarily mediated by inhibiting penicillin-binding protein 3 (PBP3), thereby disrupting bacterial cell wall synthesis (25). Furthermore, cefiderocol is highly stable against all classes of carbapenemases and mainly reserved as a last resort for the treatment of MBL-producing CRE infections (26).

However, a concerning finding in this study was that 19 (25.0%) *bla*_NDM_-harboring *K. pneumoniae* and *E. coli* isolates were non-susceptible to cefiderocol based on disk diffusion testing. This aligns with recent reports from China and India documenting cefiderocol-resistant *bla*_NDM_ -producing Enterobacterales (27, 28). Given that cefiderocol has not yet been utilized at the two studied centers, this indicates that the resistance may be emerging independently of prior exposure. A few studies (29, 30) have suggested that mutations in the *cirA* gene (such as truncation or premature stop codons) could contribute to cefiderocol resistance in *bla*_NDM_ -producing CRE isolates without prior exposure. To the best of our knowledge, this is the first study to report cefiderocol resistance in *bla*_NDM_ -producing CRE isolates in Southeast Asia.

Ceftazidime-avibactam and imipenem-cilastatin-relebactam are recently approved β-lactam/β-lactamase inhibitor combinations with distinct activity profiles depending on the carbapenemase present. Avibactam, a novel β-lactamase inhibitor, is effective against clinically relevant class A and some class D carbapenemases. In this study, among 17 isolates harboring *bla*_OXA-48-like_, ceftazidime-avibactam demonstrated 70.6% susceptibility, second only to cefiderocol (100%). A previous study (31) reported higher ceftazidime-avibactam susceptibility rates (98.9%) against *bla*_OXA-48-like_-producing isolates.

Conversely, imipenem-cilastatin-relebactam, while highly effective against KPC-producing strains, showed limited activity against oxacillinase-producing isolates, with only 17.6% susceptibility in this study. This is due to relebactam’s poor inhibition of OXA-48-like enzymes, making this agent unsuitable for treating oxacillinase-producing CRE infections, regardless of in vitro susceptibility (32). Although rare cases of *bla*_KPC_ have been reported in Malaysia (11), none were detected in this study, emphasizing the low prevalence of this carbapenemase in the region. Consequently, imipenem-cilastatin-relebactam holds limited clinical relevance in Malaysian healthcare settings.

To date, there is no β-lactam/β-lactamase inhibitor combinations approved by the United States Food and Drug Administration (FDA) currently targeting MBL-producing *Enterobacterales*, despite several promising agents being in development (6). Another promising treatment option for MBL-producing isolates is the combination of ceftazidime-avibactam and aztreonam. Aztreonam, a monocyclic β-lactam antibiotic, remains unaffected by MBL carbapenemases but is susceptible to hydrolysis by other β-lactamases, including extended-spectrum β-lactamases (ESBLs), AmpC cephalosporinases, KPCs, and OXA-48-like enzymes, many of which are co-produced by MBL-producing CRE isolates. In this combination, avibactam effectively inhibits these additional β-lactamases, restoring aztreonam’s activity.

In this study, the combination of ceftazidime-avibactam and aztreonam demonstrated an in vitro synergic activity against all isolates harboring MBL-producing genes, as assessed by the broth disk elution method. This finding agrees with a large-scale study evaluating the *in vitro* activity of aztreonam/avibactam against 1,098 clinical CRE isolates collected from hospitals across Europe, Asia, and Latin America, and reported 99.6% overall susceptibility including 100% susceptibility among MBL-producing isolates (33).

A total of 52 (33.8%) CRE isolates were categorized as non-CP-CRE due to the absence of the five tested carbapenemase-producing genes. As whole-genome sequencing was not performed due to financial constraints, the precise mechanisms of carbapenem resistance in these isolates remain unclear. Moreover, representative isolates from this group tested negative using the Xpert Carba-R assay. Carbapenem resistance in non-CP-CRE may result from β-lactamase activity combined with structural mutations. The production of ESBLs, typically plasmid-encoded or the hyperproduction of AmpC from inducible or derepressed chromosomal genes, in combination with porin mutations, can decrease outer membrane permeability, reducing antibiotic diffusion across the bacterial membrane (4). Other resistance mechanisms in non-CP-CRE include overexpression of drug efflux pumps, alterations in penicillin-binding proteins, and modifications in biofilm components (19). However, the possibility that these isolates harbor carbapenemase genes not included in our testing cannot be excluded, representing a limitation of this study.

Other limitations of this study include the fact that AST for cefiderocol susceptibility testing, whether by disk diffusion or broth microdilution, can be significantly influenced by factors, such as iron concentration and inoculum size. To enhance accuracy and reduce the risk of false results, it is recommended to test subsequent isolates (9). Despite these limitations, the findings presented offer valuable insights and represent an important step toward the tailored management of CRE-producing infections.

Based on the present study, the *bla*_NDM_ gene is the most prevalent carbapenemase-producing gene identified, while cefiderocol shows the greatest activity compared with other novel β-lactams tested against CRE isolates. Two novel β-lactamase inhibitor combination agents, ceftazidime-avibactam and imipenem-cilastatin-relebactam, demonstrated poor activity against the predominant NDM-producing CRE isolates but showed relatively better efficacy against OXA-48-like producers. Alarmingly, resistance to cefiderocol has been observed in NDM-producing CRE isolates even without prior drug exposure. The synergistic combination of ceftazidime-avibactam plus aztreonam is another promising treatment option for MBL-producing isolates apart from cefiderocol. This study underscores the critical need for molecular identification of carbapenemase-producing genes in CRE isolates, as this plays a key role in selecting the most effective novel β-lactam agent for use in healthcare settings.
